# Dietary Total Prenylflavonoids from the Fruits of *Psoralea corylifolia* L. Prevents Age-Related Cognitive Deficits and Down-Regulates Alzheimer’s Markers in SAMP8 Mice

**DOI:** 10.3390/molecules23010196

**Published:** 2018-01-18

**Authors:** Zhi-Jing Chen, Yan-Fang Yang, Ying-Tao Zhang, Dong-Hui Yang

**Affiliations:** School of Pharmaceutical Sciences, Peking University, Beijing 100191, China; czhijing@163.com (Z.-J.C.); yangyanfang@bjmu.edu.cn (Y.-F.Y.); ydhui@bjmu.edu.cn (D.-H.Y.)

**Keywords:** Alzheimer’s disease, *Psoralea corylifolia*, prenylflavonoids, resveratrol, SAMP8

## Abstract

Alzheimer’s disease (AD) is a serious threat for the aging society. In this study, we examined the preventive effect of the total prenylflavonoids (TPFB) prepared from the dried fruits of *Psoralea corylifolia* L., using an age-related AD mouse model SAMP8. We found that long-term dietary TPFB at 50 mg/kg·day significantly improved cognitive performance of the SAMP8 mice in Morris water maze tests, similar to 150 mg/kg·day of resveratrol, a popular neuro-protective compound. Furthermore, TPFB treatment showed significant improvements in various AD markers in SAMP8 brains, which were restored to near control levels of the normal mice, SAMR1. TPFB significantly reduced the level of amyloid β-peptide 42 (Aβ42), inhibited hyperphosphorylation of the microtubule-associated protein Tau, induced phosphorylation of Ser9 of the glycogen synthase kinase 3β (GSK-3β), and decreased the expression of the proinflammatory cytokines TNFα, IL-6, and IL-1β. Finally, TPFB also markedly reduced the level of serum derivatives of reactive oxygen metabolites (d-ROMs), a biomarker of oxidative stress in vivo. These results showed that dietary TPFB could effectively prevent age-related cognitive deficits and AD-like neurobiochemical changes, and may have a potential role in the prevention of Alzheimer’s disease.

## 1. Introduction

Alzheimer’s disease (AD) is a fatal neurodegenerative brain disease and the most common cause of dementia, affecting about 47 million people worldwide [1]. The prevalence of AD is aging-associated and primarily seen in the senile period. AD is one of the top 10 leading causes of death among adults aged 65 years or older [2]. Unfortunately, currently no available treatments can stop, reverse, or even slow the progression of AD, stressing the urgent need for AD drug development. The etiology and pathogenesis of AD are very complex and remain unclear. There are two core pathological changes found in the brains of AD patients: the extracellular amyloid plaques formed by the aggregation and deposition of amyloid β-peptide (Aβ) [3,4], and the neurofibrillary tangles—the aggregates of the microtubule-associated protein Tau that is abnormally hyperphosphorylated and accumulates inside neuronal cells [5,6]. It has been suggested that toxic oligomers generated during the aggregation of Aβ and Tau are mainly responsible for irreversible neuronal death and loss [7]. However, other mechanisms such as neuroinflammation and oxidative stress may be involved as well, which have drawn more attention in recent years [8,9].

Buguzhi or Bakuchi—the dried fruit of *Psoralea corylifolia* L.—is a well-known herb commonly used in traditional Chinese and Ayurvedic medicine for various conditions such as psoriasis, vitiligo, hair loss, impotence, osteoporosis, etc. [10,11]. The herb is also used as a kidney tonic in Chinese herbal formulas to treat senile dementia. Previously, we and others have found that several individual prenylflavonoids from Buguzhi exhibited in vitro biological activities targeting Aβ production and aggregation [12,13]. However, none of these prenylflavonoids has ever been tested in an AD-related animal model, likely due to their low contents in the plant.

In this study, we have established a simple method to enrich the total prenylflavonoids (TPFB) from Buguzhi, and further explored the beneficial effects and the possible mechanisms of long-term dietary TPFB in the senescence-accelerated-mouse prone 8 (SAMP8), an age-related AD model sharing many biological features to AD [14,15,16,17].

## 2. Results

### 2.1. Yield and Chemical Composition of TPFB

**Figure 1:**
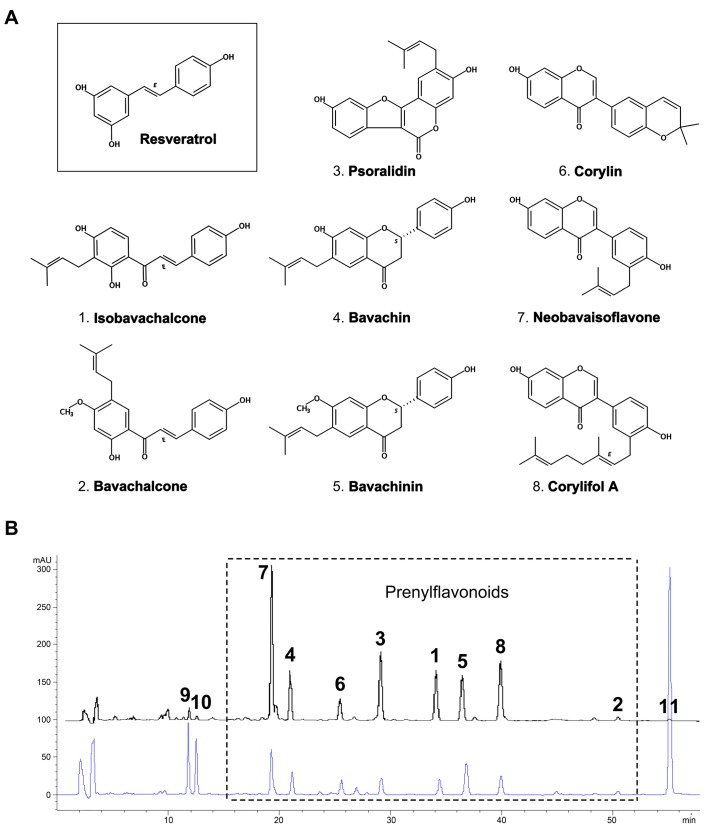
(**A**) Chemical structures of resveratrol and eight major compounds in total prenylflavonoids (TPFB); and (**B**) a representative HPLC chromatograph of TPFB (upper line) in comparison with the methanol extract of Buguzhi (lower line) detected at wavelength 245 nm. Peaks: (**1**) Isobavachalcone; (**2**) Bavachalcone; (**3**) Psoralidin; (**4**) Bavachin; (**5**) Bavachinin; (**6**) Corylin; (**7**) Neobavaisoflavone; (**8**) Corylifol A; (**9**) Psoralen; (**10**) Isopsoralen; (**11**) Bakuchiol. Note: (**9**), (**10**) and (**11**) are major compounds in Buguzhi but are successfully removed in TPFB.

The current protocol applied a double-defatting strategy followed by activated carbon purification, which enabled us to prepare a prenylflavonoid-enriched extract TPFB from Buguzhi with an average yield of 1.92%. Eight major components (Figure 1A) were quantified by HPLC as isobavachalcone (**1**) 10.42%; bavachalcone (**2**) 2.08%; psoralidin (**3**) 7.95%; bavachin (**4**) 14.98%; bavachinin (**5**) 5.38%; corylin (**6**) 1.83%; neobavaisoflavone (**7**) 14.99%; and corylifol A (**8**) 8.52%, respectively. They roughly accounted for 66% of the total weight of TPFB. As shown in Figure 1B, the preparation method efficiently removed three abundant but non-target compounds including psoralen (0.76%), isopsoralen (0.22%), and bakuchiol (0.76%).

### 2.2. TPFB Prevented Age-Related Cognitive Impairments of SAMP8 Mice

Next, we treated SAMP8 mice with dietary TPFB for 9 months to determine its preventive effects on the cognitive deficits seen in aged SAMP8 mice, using resveratrol as a positive control. Thirty male SAMP8 and ten male SAMR1 mice of three months of age were assigned to four groups (10 for each): normal control group SAMR1, model control group SAMP8, SAMP8-RSV group (resveratrol, 150 mg/kg·day), and SAMP8-TPFB group (TPFB, 50 mg/kg·day).

**Figure 2:**
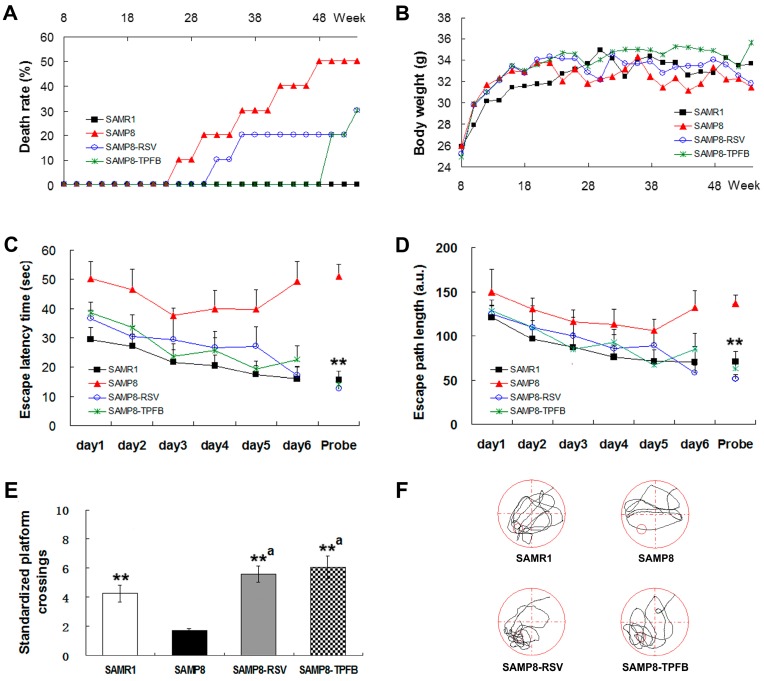
Dietary TPFB significantly improved the spatial learning and memory performance of SAMP8 mice. During the experiment, the SAMP8 group showed increased death rate whereas no death was observed in SAMR1 mice. Both TPFB (50 mg/kg·day) and resveratrol (RSV, 150 mg/kg·day) groups showed (**A**) lower death rates and (**B**) slightly higher body weights. At the age of 12 months, SAMP8 mice (*n* = 5) displayed behavioral impairments in Morris water maze (MWM) test with significantly (**C**) longer escape latency time and (**D**) longer escape path length than SAMR1 (*n* = 10) during training and probe trials, and they accomplished the probe test with (**E**) fewer standardized platform crossings and (**F**) abnormal path traces. Nine-month treatment by TPFB (50 mg/kg·day, *n* = 7) or resveratrol (RSV, 150 mg/kg·day, *n* = 7) significantly improved above parameters of SAMP8 to near-normal levels of SAMR1. Data are expressed as mean ± SEM. ** *p* < 0.01 vs. SAMP8 group; a Non-significant vs. SAMR1 group.

During the experiment, no drug-related toxic effects were found either in the SAMP8-RSV or the SAMP8-TPFB group. However, we observed some natural deaths in SAMP8 mice but not in SAMR1: five deaths in the SAMP8 group, three deaths in the SAMP8-RSV group and three deaths in the SAMP8-TPFB group (Figure 2A). No significant difference was found among groups in food and water intake, although the SAMP8 group exhibited slightly lower body-weight at the late stage of the experiment (Figure 2B).

We used the Morris water maze (MWM) test to assess the spatial recognition memory performance of the mice at the age of 12 months. As seen in Figure 2C, SAMP8 mice showed impaired spatial learning strategies in the training days and they performed poorly in the probe test as well. The escape latency time of the SAMP8 group decreased during the first three training days and increased thereafter. In contrast, the other three groups all accomplished the task with steadily enhanced spatial learning acquisition during training. Similar results were obtained in the probe test in which the SAMP8 group spent far more time reaching the platform area, suggesting impaired spatial memory. To exclude the influence of the swimming speed, the average escape path length was also analyzed for each group and significant differences were likewise observed between the SAMP8 group and the other three groups (Figure 2D). The SAMP8 group also achieved significantly fewer platform crossings (standardized by swim distance) in the probe test compared to the other groups (Figure 2E). Path trace analysis revealed that SAMP8 mice tended to navigate more or less aimlessly in the probe test (Figure 2F). Overall, both TPFB and resveratrol treatment markedly improved the behavioral performance of the SAMP8 mice in the MWM test, demonstrating their beneficial effects on spatial memory and cognitive functions.

### 2.3. TPFB Reduced Aβ42 Accumulation, Tau Phosphorylation, and Glycogen Synthase Kinase 3β (GSK-3β) Activation in SAMP8 Brains

It has been well documented that aged SAMP8 mice show various neurobiochemical alterations in their brains sharing similar features to AD. To determine the possible effects of TPFB on these AD markers, all mice were sacrificed and their brain tissues were collected for biochemical analysis.

**Figure 3:**
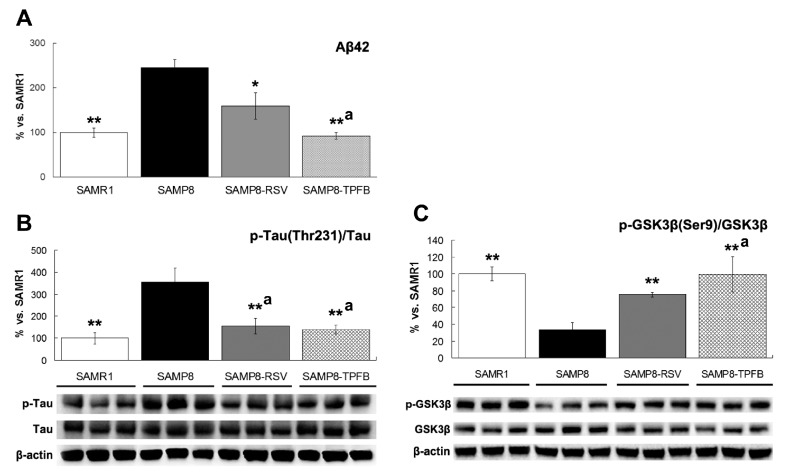
TPFB significantly decreased protein levels of Aβ42 and Thr231-phosphorylated Tau, and increased Ser9-phosphorylated glycogen synthase kinase 3β (GSK-3β) in SAMP8 mice brains. (**A**) Aβ42 was analyzed by enzyme-linked immunosorbent assay (ELISA); (**B**) Tau and (**C**) GSK-3β were measured by Western blots. Compared to SAMR1 (*n* = 10), SAMP8 mice brains (*n* = 5) showed significant overproduction of Aβ42, hyperphosphorylation of Tau (Thr231), and hypophosphorylation of GSK-3β (Ser9) at the age of 12 months, whereas TPFB (50 mg/kg·day, *n* = 7) and resveratrol (RSV, 150 mg/kg·day, *n* = 7) significantly attenuated these changes. Data are expressed as mean ± SEM. * *p* < 0.05, ** *p* < 0.01 vs. SAMP8 group; a Non-significant vs. SAMR1 group.

Accumulation and aggregation of the amyloid peptide (mostly Aβ42), as well as hyperphosphorylation of Tau are two hallmarks of AD brains. Consistent with previous report [18], 12 month-old SAMP8 mice showed a significantly higher amount of Aβ42 and phosphorylated Tau (Thr231) in their brains than SAMR1, and treatment with either resveratrol or TPFB significantly reduced the level of Aβ42 and down-regulated the phosphorylation of Tau (Figure 3A,B). GSK-3β is the primary tau kinase, and dephosphorylation of the Serine at position 9 is required for its activity [19]. Compared to SAMR1, the SAMP8 mice showed a significantly lower level of Ser9 phosphorylation of GSK-3β, indicating overactivation of GSK-3β. Treatment of SAMP8 mice with either resveratrol or TPFB significantly enhanced the Ser9 phosphorylation of GSK-3β (Figure 3C).

### 2.4. TPFB Decreased Brain Expression of TNF-α, IL-6, and IL-1β in SAMP8 Mice

**Figure 4:**
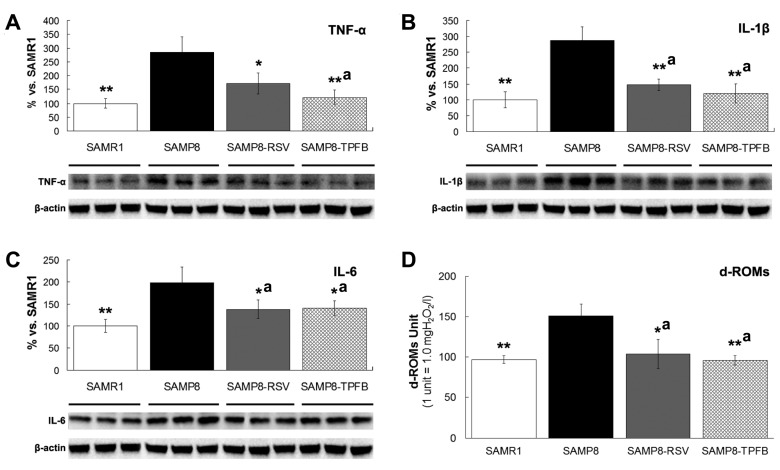
TPFB significantly decreased the expression of proinflammatory cytokines in SAMP8 mice brains and reduced oxidative levels in SAMP8 mice sera. (**A**) TNF-α; (**B**) IL-1β; and (**C**) IL-6 were measured by Western blots; (**D**) Serum oxidative levels were measured by derivatives of reactive oxygen metabolites (d-ROMs) test. Compared to SAMR1 (*n* = 10), SAMP8 mice (*n* = 5) produced significantly more TNF-α, IL-1β, and IL-6 in their brains and d-ROMs in their sera at the age of 12 months, whereas TPFB (50 mg/kg·day, *n* = 7) and resveratrol (RSV, 150 mg/kg·day, *n* = 7) significantly restored these values to the normal range of SAMR1. Data are expressed as mean ± SEM. * *p* < 0.05, ** *p* < 0.01 vs. SAMP8 group; a Non-significant vs. SAMR1 group.

Chronic neuroinflammation is a common feature shared by AD and SAMP8 brains. As shown in Figure 4, protein levels of the proinflammatory cytokines TNF-α, IL-1β, and IL-6 were significantly higher in SAMP8 brains compared to SAMR1, and both TPFB and resveratrol treatment attenuated this elevation dramatically (Figure 4A–C), demonstrating their anti-neuroinflammatory activities in vivo.

### 2.5. TPFB Reduced Serum Oxidative Level in SAMP8 Mice

Oxidative stress is also considered to play a significant role in the pathogenesis of AD. Hence, we collected the serum from each animal and carried out a d-ROMs (derivatives of reactive oxygen metabolites) test to measure the whole-body oxidative level of the mice. As expected, a significantly higher level of oxidative states was observed in SAMP8 control mice, and treatment with either TPFB or resveratrol considerably reduced the serum d-ROMs of SAMP8 to normal levels of SAMR1 (Figure 4D).

## 3. Discussion

Prenylflavonoids or prenylated flavonoids are a sub-class of flavonoids sharing a common feature of a prenyl group attached to the flavonoid backbones including chalcones, flavones, flavanones, and flavonols. Prenylflavonoids are usually more hydrophobic and more affinitive to target proteins in comparison to their flavonoid analogues, which may endow them with better bioactivities and bioavailabilities [20]. Various bioactivities such as estrogenic, immunomodulatory, anti-antioxidative, anti-microbial, and anticancer activity have been reported for prenylflavonoids [21], but only a few dealt with the neuroprotective effects. In this study, we found that long-term dietary TPFB—a purified extract from Buguzhi containing over 66% of prenylflavonoids—showed strong preventive effects on age-related cognitive deficits and neurobiochemical alterations in SAMP8 mice.

Consistent with previous studies [18,22,23], dietary resveratrol displayed strong beneficial effects both on behavioral performance and AD biomarkers of SAMP8 mice in this study. The in vivo neuroprotective effects of resveratrol have also been reported in normal aging mouse model [24], scopolamine-induced mouse/rat model [25,26], and transgenic mouse models such as Tg2576 and APP/PS1 [27]. Recently, the results of two phase 2 clinical trials have been released, demonstrating preliminary positive effects of resveratrol in mild–moderate AD patients [28,29].

**Figure 5:**
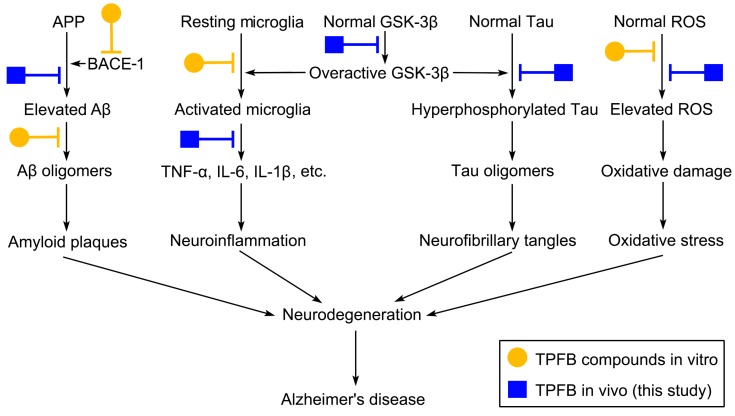
Schematic diagram illustrating major Alzheimer’s disease (AD) pathological pathways and the potential targets of TPFB in this study and its major components in previous studies [12,13,30,31,32,33,34]. ROS: reactive oxygen species. Aβ: amyloid β-peptide.

It is noteworthy that TPFB, as a multi-compound mixture, achieved quite similar or even stronger beneficial effects in vivo at a much lower dose (50 mg/kg·day) in comparison with resveratrol (150 mg/kg·day). A possible explanation of this phenomenon might be the potential synergistic and multi-target effects of TPFB compounds (Figure 5). Previously, we and others have shown potential anti-AD effects of several major compounds of TPFB targeting multiple AD pathways. For instance, isobavachalcone and bavachinin can effectively inhibit the on-pathway aggregation of Aβ42 and attenuate Aβ42 neurotoxicity in vitro [13]; bavachalcone and bavachinin—as well as several other prenyflavonoids in TPFB—can significantly inhibit the key Aβ-producing enzyme BACE-1 in vitro [12]; isobavachacone, corylin, and neobavaisoflavone have strong in vitro and in vivo anti-inflammatory activities and reduce the expression of TNF-α, IL-6, and IL-1β [30,31,32]. TPFB compounds are also inherent antioxidants due to their phenolic properties, and have also been confirmed in several in vitro studies [33,34]. Besides, TPFB compounds possess phenolic backbones resembling resveratrol in 2D shapes (Figure 1), which may partially account for their similar effects in SAMP8.

The present study provides another piece of valuable information—that TPFB also strongly inhibited another hallmark of AD, Tau hyperphosphorylation, likely through inactivation of the key kinase GSK-3β. The negative correlation between Tau (Thr231) phosphorylation and GSK-3β (Ser9) phosphorylation was clearly shown in all groups of this study (Figure 3B,C), suggesting the critical role of GSK-3β in Tau hyperphosphorylation in SAMP8 mice brains. Consistent with this finding, a recent study reported that in 12-month-old SAMP8 mice, Tau phosphorylation level as well as the learning and memory performance could be significantly improved by direct inhibition of GSK-3β activity using antisense oligonucleotide [35]. Furthermore, conditional overexpression of GSK-3β in normal mice brains induced AD-like phenotype with tau hyperphosphorylation, apoptotic neuronal death, as well as spatial learning deficit, and these alterations could be fully reversed after restoration of normal GSK-3β levels by gene shutdown [36]. This evidence suggests that GSK-3β is not only the major player in Tau pathology, but may also be responsible for the cognitive impairments seen in SAMP8 mice. In fact, recent advances strongly support the view that GSK-3β plays a pivotal role in the etiopathogenesis of AD, linking Aβ, tau, and neuroinflammation pathways [37,38,39]. Nevertheless, it remains to be confirmed whether the beneficial effects of TPFB in SAMP8 mice are primarily due to the GSK-3β inhibitory activity. Further studies are needed to clarify the active molecules and the exact mechanism of TPFB regulating GSK-3β.

In recent years, many plant phenols such as flavonoids and stilbenes have drawn considerable attention due to their remarkable anti-AD effects both in vitro and in vivo [40]. However, most of the natural anti-AD molecules are not as potent as the synthetic ones when tested solely, largely due to their unoptimized nature or unfavorable pharmacokinetic profiles. The most conspicuous representative might be resveratrol, a popular but “paradoxical” stilbene with high potency but poor bioavailability for being rapidly and extensively metabolized and excreted [41]. Interestingly, a recent study demonstrated that semisynthetic prenylated resveratrol derivatives were 2- to 20-fold more potent than resveratrol with better anti-BACE-1, anti-Aβ aggregation, and antioxidant activities [42]. Besides, two natural prenylated resveratrol analogs arachidin-1 and -3 were found to exhibit improved glucuronidation profiles which may increase their bioavailability via slowed metabolism [43]. Accordingly, the prenyl group shared by TPFB compounds may significantly improve their druglikeness as well as the potency. Indeed, in a recent pharmacokinetic study in our lab, all TPFB compounds involved in the present study were readily detected in rat brains after an oral administration of Buguzhi extract [44].

Finally, we cannot rule out the possibility that a single powerful compound may exist in TPFB perfectly mimicking resveratrol but with much higher potency. To clarify that question, individual TPFB compounds need to be prepared and tested in the same experimental conditions. We are currently working on the large-scale isolation of individual TPFB compounds.

It should be noted that there are some limitations in this study. First, the present study was designed with a relatively limited scope, primarily focusing on AD-related pathways and potential drug targets (Figure 5), thus some important issues such as other anti-ageing or possible side effects of TPFB were not covered by the study. Second, it remains to be confirmed whether TPFB could specifically target and benefit the cognition-related brain regions such as the frontal cortex and the hippocampus. Third, according to the structural and functional similarities of TPFB compounds and resveratrol, it deserves more studies to explore their possible effects on the ageing-regulator Sirt1 [45] and important synaptic markers such as syntaxin-1, PSD95, and synaptophysin [46,47].

Taken together, in this study we found that TPFB—the total prenylflavonoids from *Psoralea corylifolia* L.—is a promising drug candidate preventing age-related cognitive impairments and AD-like neurobiochemical changes in SAMP8 mouse model, which effectively inhibited Aβ42 production, tau hyperphosphorylation, GSK-3β overactivation, proinflammatory cytokines production, as well as oxidative stress.

## 4. Materials and Methods

### 4.1. Preparation and Quantification of the Total Prenylflavonoids from Buguzhi (TPFB)

Buguzhi was purchased from Beijing Tongrentang Co., Ltd. (Beijing, China) and identified as the dried fruit of *Psoralea corylifolia* L. in Leguminosae. The fruits were pulverized into a fine power, defatted with petroleum ether, and then extracted with ethyl acetate to produce a raw extract. The extract was defatted again and further purified using activated carbon.

Eight major components in TPFB (bavachalcone, isobavachalcone, neobavaisoflavone, bavachin, bavachinin, corylin, psoralidin, and corylifol A shown in Figure 1A) were analyzed by using an established HPLC method described previously [13]; three non-target but predominant compounds in Buguzhi (psoralen, isopsoralen, and bakuchiol) were also quantified. Resveratrol (Figure 1A) and other chemical standards used in HPLC analysis were obtained from Shanghai Yuanye Bio-Technology Co. (Shanghai, China).

### 4.2. Animals and Drug Intervention

All animal experiments were carried out in accordance with the protocol approved by the Institutional Animal Care and Use Committee of Peking University Health Science Center (Beijing, China).

Thirty male SAMP8 and ten male SAMR1 mice of three months of age were assigned to four groups (10 for each): normal control group SAMR1, model control group SAMP8, SAMP8-RSV group (resveratrol, 150 mg/kg·day; positive control), SAMP8-TPFB group (TPFB, 50 mg/ kg·day). The 50 mg/kg·day of TPFB for mice is roughly equivalent to about 15 g/day of Buguzhi consumption for a human being, which is a regular dosage used in traditional Chinese medicine. The dosage of resveratrol was set according to the previous reports [18,23]. The drugs were dispersed in a small amount of honey water and administered to the mice once daily. The mice were trained to voluntarily drink 0.5 mL of the drug-honey water, which was manually fed with a dropper. The SAMR1 and SAMP8 control group received same amount of the honey water. All animals were fed with a standard diet and were kept in the same housing room, maintained at a temperature of 22 ± 2 °C, a relative humidity of 50 ± 10%, and a 12-h light/dark automatic light cycle.

### 4.3. Morris Water Maze Test (MWM)

The Morris water maze consisted of a metal pool (120 cm in diameter) filled with tap water (25 °C), and a removable escape platform, hidden 1 cm beneath the surface of water. The experiment consisted of a 6-day acquisition followed by a probe test on day 7. In the acquisition trials, animals were trained to memorize the platform location in relation to distinct cues painted on the inner wall and the surrounding white curtain. Each mouse took three training trials daily at different start points with an intertrial interval of 20 min. The animals were gently placed in the water facing the wall of the pool and allowed to swim to find the platform. If the mice failed to find the platform in 60 s, they were guided to the platform by the experimenter. The time for animals to climb up the hidden platform was recorded as escape latency. In the probe trial, the platform was removed and each mouse was allowed to swim for 120 s. Several measures were used for memory and learning assessment including escape latency, escape path length, platform crossings, and path traces.

### 4.4. Blood and Brain Tissue Collection

The mice were sacrificed after behavioral tests at the age of 12 months. Blood samples were collected from the orbital sinus, left undisturbed at room temperature for 1 h, and centrifuged at 3000 rpm for 15 min at 4 °C. The serum was collected and stored at −80 °C until use.

The whole mouse brain was quickly removed after blood collection, bisected sagittally on an ice plate, then frozen by liquid nitrogen separately and stored at −80 °C until protein extraction.

### 4.5. Western Blot

Brain tissue samples for Western blot were homogenized by using Mini-Bead Beater-16 (BIOSPEC, Bartlesville, OK, USA) in pre-cold lysis buffer (50 mM Tris-HCl, 150 mM NaCl, 5 mM EDTA, 1% Triton X-100, pH 7.4) containing Phosphatase Inhibitor Cocktail (Roche, Switzerland) and Sigma FAST Protease Inhibitor Tablet (Sigma-Aldrich, St. Louis, MO, USA). The homogenate was placed on ice for 30 min with gentle rocking, then centrifuged at 10,000× *g* for 10 min at 4 °C to collect supernatant. Protein concentration was determined using Pierce BCA Protein Assay Kit (Thermo Scientific, Waltham, MA, USA) according to manufactory protocol. About 30 μg of denatured protein were separated on a 4–20% SDS-PAGE gel (Genscript, Piscataway, NJ, USA) and transferred to an Immobilon polyvinylidene difluoride membrane (Millipore, Billerica, MA, USA). The membranes were incubated overnight at 4 °C with the primary antibodies (Abcam, Cambrige, UK) diluted in Tris-buffered saline containing 0.1% Tween 20 (TBS-T) and 5% BSA. Membranes were then washed and blotted with secondary antibodies (Abcam, Cambrige, UK) in TBS-T for 1 h at room temperature. Protein bands were visualized using a chemiluminescence detection kit (Clarity Western ECL Substrate (BIO-RAD, ‎Hercules, CA, USA). Band intensities were quantified using Image Lab (Bio-Rad).

### 4.6. ELISA

Brain tissue samples for Aβ42 ELISA were thoroughly homogenized by Mini-Bead Beater-16 in pre cold buffer (5 M Guanidine HCl, 50 mM Tris HCl, pH = 8.0) containing the Phosphatase Inhibitor Cocktail and Sigma FAST Protease Inhibitor Tablet, and incubated at room temperature for 4 h with gentle rocking. The samples were then centrifuged at 10,000× *g* for 10 min at 4 °C and the supernatants were collected. Protein concentration was determined as mentioned above. The protein samples were diluted to 300 μg/mL of total protein and Aβ42 levels were assessed using SensoLyte^®^ Anti-Mouse/Rat β-Amyloid (1–42) Quantitative ELISA kit (AnaSpec, Fremont, CA, USA).

### 4.7. Serum d-ROMs Test

The derivatives of reactive oxygen metabolites (d-ROMs) in serum were measured using indirect quantification of the organic hydroperoxides as described elsewhere [48]. The iron cation in an acidic buffer catalyzes the cleavage of organic hydroperoxides in the serum, leading to the generation of free radicals, and the free radicals convert DEPPD (*N*,*N*-diethyl-para-phenylendiamine) into a red substance, which can be measured by a spectrophotometric plate reader at 505 nm. Hydrogen peroxide was used as a standard reference to generate a calibration curve. The results are expressed as d-ROMs units equivalent to levels of hydrogen peroxide (1 unit = 1.0 mg H_2_O_2_/L).

### 4.8. Statistical Analysis

The results were statistically analyzed using Microsoft Excel 2013. Data are expressed as the means ± SEM, and the means were compared with ANOVA. Statistical significance was considered when *p*-values were <0.05.

## References

[1] Bronzuoli M.R., Iacomino A., Steardo L., Scuderi C. (2016). Targeting neuroinflammation in Alzheimer’s disease. J. Inflamm. Res..

[2] Alzheimer’s Association (2017). Alzheimer’s disease facts and figures. Alzheimer’s Dement..

[3] Funato H., Yoshimura M., Kusui K., Tamaoka A., Ishikawa K., Ohkoshi N., Namekata K., Okeda R., Ihara Y. (1998). Quantitation of amyloid beta-protein (A beta) in the cortex during aging and in Alzheimer’s disease. Am. J. Pathol..

[4] Tam J.H., Pasternak S.H. (2012). Amyloid and Alzheimer’s disease: Inside and out. Can. J. Neurol. Sci..

[5] Kosik K.S., Joachim C.L., Selkoe D.J. (1986). Microtubule-associated protein tau (tau) is a major antigenic component of paired helical filaments in Alzheimer disease. Proc. Natl. Acad. Sci. USA.

[6] Šimić G., BabićLeko M., Wray S., Harrington C., Delalle I., Jovanov-Milošević N., Bažadona D., Buée L., de Silva R., Di Giovanni G. (2016). Tau protein hyperphosphorylation and aggregation in Alzheimer’s disease and other tauopathies, and possible neuroprotective Strategies. Biomolecules.

[7] Lv Z.Y., Tan C.C., Yu J.T., Tan L. (2017). Spreading of pathology in Alzheimer’s disease. Neurotox. Res..

[8] Bagyinszky E., Giau V.V., Shim K., Suk K., An S.S.A., Kim S. (2017). Role of inflammatory molecules in the Alzheimer’s disease progression and diagnosis. J. Neurol. Sci..

[9] Tönnies E., Trushina E. (2017). Oxidative stress, synaptic dysfunction, and Alzheimer’s disease. J. Alzheimer’s Dis..

[10] Chopra B., Dhingra A.K., Dhar K.L. (2013). *Psoralea corylifolia* L. (Buguchi)—Folklore to modern evidence: Review. Fitoterapia.

[11] Zhang X., Zhao W., Wang Y., Lu J., Chen X. (2016). The chemical constituents and bioactivities of *Psoralea corylifolia* Linn.: A review. Am. J. Chin. Med..

[12] Choi Y.H., Yon G.H., Hong K.S., Yoo D.S., Choi C.W., Park W.K., Kong J.Y., Kim Y.S., Ryu S.Y. (2008). In vitro BACE-1 inhibitory phenolic components from the seeds of *Psoralea corylifolia*. Planta Med..

[13] Chen X., Yang Y., Zhang Y. (2013). Isobavachalcone and bavachinin from *Psoraleae* Fructus modulate Aβ42 aggregation process through different mechanisms in vitro. FEBS Lett..

[14] Pallas M., Camins A., Smith M.A., Perry G., Lee H.G., Casadesus G. (2008). From aging to Alzheimer’s disease: Unveiling “the switch” with the senescence-accelerated mouse model (SAMP8). J. Alzheimer’s Dis..

[15] Morley J.E., Armbrecht H.J., Farr S.A., Kumar V.B. (2012). The senescence accelerated mouse (SAMP8) as a model for oxidative stress and Alzheimer’s disease. Biochim. Biophys. Acta.

[16] Morley J.E., Farr S.A., Kumar V.B., Armbrecht H.J. (2012). The SAMP8 mouse: A model to develop therapeutic interventions for Alzheimer’s disease. Curr. Pharm. Des..

[17] Cheng X.R., Zhou W.X., Zhang Y.X. (2014). The behavioral, pathological and therapeutic features of the senescence-accelerated mouse prone 8 strain as an Alzheimer’s disease animal model. Ageing Res. Rev..

[18] Porquet D., Casadesús G., Bayod S., Vicente A., Canudas A.M., Vilaplana J., Pelegrí C., Sanfeliu C., Camins A., Pallàs M. (2013). Dietary resveratrol prevents Alzheimer’s markers and increases life span in SAMP8. AGE.

[19] Beurel E., Grieco S.F., Jope R.S. (2015). Glycogen synthase kinase-3 (GSK3): Regulation, actions, and diseases. Pharmacol. Ther..

[20] Botta B., Menendez P., Zappia G., de Lima R.A., Torge R., Monachea G.D. (2009). Prenylated isoflavonoids: Botanical distribution, structures, biological activities and biotechnological studies. An update (1995–2006). Curr. Med. Chem..

[21] Yang X., Jiang Y., Yang J., He J., Sun J., Chen F., Zhang M., Yang B. (2015). Prenylated flavonoids, promising nutraceuticals with impressive biological activities. Trends Food Sci. Technol..

[22] Ginés C., Cuesta S., Kireev R., García C., Rancan L., Paredes S.D., Vara E., Tresguerres J.A. (2017). Protective effect of resveratrol against inflammation, oxidative stress and apoptosis in pancreas of aged SAMP8 mice. Exp. Gerontol..

[23] Palomera-Ávalos V., Griñán-Ferré C., Puigoriol-Ilamola D., Camins A., Sanfeliu C., Canudas A.M., Pallàs M. (2017). Resveratrol protects SAMP8 brain under metabolic stress: Focus on mitochondrial function and Wnt Pathway. Mol. Neurobiol..

[24] Palomera-Ávalos V., Griñán-Ferré C., Izquierdo V., Camins A., Sanfeliu C., Pallàs M. (2017). Metabolic stress induces cognitive disturbances and inflammation in aged mice: Protective role of resveratrol. Rejuvenation Res..

[25] Gacar N., Mutlu O., Utkan T., Celikyurt I.K., Gocmez S.S., Ulak G. (2011). Beneficial effects of resveratrol on scopolamine but not mecamylamine induced memory impairment in the passive avoidance and Morris water maze tests in rats. Pharmacol. Biochem. Behav..

[26] Gupta R., Gupta L.K., Mediratta P.K., Bhattacharya S.K. (2012). Effect of resveratrol on scopolamine-induced cognitive impairment in mice. Pharmacol. Rep..

[27] Ma T., Tan M.S., Yu J.T., Tan L. (2014). Resveratrol as a therapeutic agent for Alzheimer’s disease. Biomed. Res. Int..

[28] Turner R.S., Thomas R.G., Craft S., van Dyck C.H., Mintzer J., Reynolds B.A., Brewer J.B., Rissman R.A., Raman R., Aisen P.S. (2015). A randomized, double-blind, placebo-controlled trial of resveratrol for Alzheimer disease. Neurology.

[29] Moussa C., Hebron M., Huang X., Ahn J., Rissman R.A., Aisen P.S., Turner R.S. (2017). Resveratrol regulates neuro-inflammation and induces adaptive immunity in Alzheimer’s disease. J. Neuroinflamm..

[30] Jing H., Wang S., Wang M., Fu W., Zhang C., Xu D. (2017). Isobavachalcone attenuates MPTP-induced Parkinson’s disease in mice by inhibition of microglial activation through NF-κB Pathway. PLoS ONE.

[31] Hung Y.L., Fang S.H., Wang S.C., Cheng W.C., Liu P.L., Su C.C., Chen C.S., Huang M.Y., Hua K.F., Shen K.H. (2017). Corylin protects LPS-induced sepsis and attenuates LPS-induced inflammatory response. Sci. Rep..

[32] Szliszka E., Skaba D., Czuba Z.P., Krol W. (2011). Inhibition of inflammatory mediators by neobavaisoflavone in activated RAW264.7 macrophages. Molecules.

[33] Xiao G., Li G., Chen L., Zhang Z., Yin J.J., Wu T., Cheng Z., Wei X., Wang Z. (2002). Isolation of antioxidants from *Psoralea corylifolia* fruits using high-speed counter-current chromatography guided by thin layer chromatography-antioxidant autographic assay. J. Chromatogr. A.

[34] Haraguchi H., Inoue J., Tamura Y., Mizutani K. (2002). Antioxidative components of *Psoralea corylifolia* (Leguminosae). Phytother. Res..

[35] Farr S.A., Ripley J.L., Sultana R., Zhang Z., Niehoff M.L., Platt T.L., Murphy M.P., Morley J.E., Kumar V., Butterfield D.A. (2014). Antisense oligonucleotide against GSK-3β in brain of SAMP8 mice improves learning and memory and decreases oxidative stress: Involvement of transcription factor Nrf2 and implications for Alzheimer disease. Free Radic. Biol. Med..

[36] Engel T., Hernández F., Avila J., Lucas J.J. (2006). Full reversal of Alzheimer’s disease-like phenotype in a mouse model with conditional overexpression of glycogen synthase kinase-3. J. Neurosci..

[37] Llorens-Martín M., Jurado J., Hernández F., Avila J. (2014). GSK-3β, a pivotal kinase in Alzheimer disease. Front. Mol. Neurosci..

[38] Maqbool M., Mobashir M., Hoda N. (2016). Pivotal role of glycogen synthase kinase-3: A therapeutic target for Alzheimer’s disease. Eur. J. Med. Chem..

[39] Gameiro I., Michalska P., Tenti G., Cores Á., Buendia I., Rojo A.I., Georgakopoulos N.D., Hern#xE1ndez-Guijo J.M., Teresa Ramos M., Wells G. (2017). Discovery of the first dual GSK3β inhibitor/Nrf2 inducer. A new multitarget therapeutic strategy for Alzheimer’s disease. Sci. Rep..

[40] Omar S.H., Scott C.J., Hamlin A.S., Obied H.K. (2017). The protective role of plant biophenols in mechanisms of Alzheimer’s disease. J. Nutr. Biochem..

[41] Tomé-Carneiro J., Larrosa M., González-Sarrías A., Tomás-Barberán F.A., García-Conesa M.T., Espín J.C. (2013). Resveratrol and clinical trials: The crossroad from in vitro studies to human evidence. Curr. Pharm. Des..

[42] Puksasook T., Kimura S., Tadtong S., Jiaranaikulwanitch J., Pratuangdejkul J., Kitphati W., Suwanborirux K., Saito N., Nukoolkarn V. (2017). Semisynthesis and biological evaluation of prenylated resveratrol derivatives as multi-targeted agents for Alzheimer’s disease. J. Nat. Med..

[43] Brents L.K., Medina-Bolivar F., Seely K.A., Nair V., Bratton S.M., Nopo-Olazabal L., Patel R.Y., Liu H., Doerksen R.J., Prather P.L. (2012). Natural prenylated resveratrol analogs arachidin-1 and -3 demonstrate improved glucuronidation profiles and have affinity for cannabinoid receptors. Xenobiotica.

[44] Yang Y.F., Zhang Y.B., Chen Z.J., Zhang Y.T., Yang X.W. (2018). Plasma pharmacokinetics and cerebral nuclei distribution of major constituents of *Psoraleae Fructus* in rats after oral administration. Phytomedicine.

[45] Ahmed T., Javed S., Javed S., Tariq A., Šamec D., Tejada S., Nabavi S.F., Braidy N., Nabavi S.M. (2017). Resveratrol and Alzheimer’s Disease: Mechanistic Insights. Mol. Neurobiol..

[46] Wang J., Yuan J., Pang J., Ma J., Han B., Geng Y., Shen L., Wang H., Ma Q., Wang Y. (2016). Effects of Chronic Stress on Cognition in Male SAMP8 Mice. Cell. Physiol. Biochem..

[47] Tong J.J., Chen G.H., Wang F., Li X.W., Cao L., Sui X., Tao F., Yan W.W., Wei Z.J. (2015). Chronic acarbose treatment alleviates age-related behavioral and biochemical changes in SAMP8 mice. Behav. Brain Res..

[48] Hayashi I., Morishita Y., Imai K., Nakamura M., Nakachi K., Hayashi T. (2007). High-throughput spectrophotometric assay of reactive oxygen species in serum. Mutat. Res..

